# Effects of Parent-Adolescent Relationship Quality on Youth Symptoms Amidst COVID-19

**DOI:** 10.3390/bs15070862

**Published:** 2025-06-25

**Authors:** Frances M. Lobo, Casandra J. Gomez Alvarado, Giselle De Leon, Valerie V. Salcido, Paula Sanchez-Hernandez, Gabriela L. Stein

**Affiliations:** 1 Department of Human Development and Family Sciences, The University of Texas at Austin, Austin, TX 78712, USA; gabriela.stein@austin.utexas.edu; 2 Department of Psychology, The University of Texas at Austin, Austin, TX 78712, USA; cgomezalvarado@utexas.edu; 3 School of Education, Texas A&M University, College Station, TX 77843, USA; gisd23@tamu.edu; 4 Department of Psychology, UNC Greensboro, Greensboro, NC 27402, USA; vvsalcido@uncg.edu (V.V.S.); pgsanche@uncg.edu (P.S.-H.)

**Keywords:** COVID-19, parent-adolescent relationship quality, anxiety, depression

## Abstract

Amidst the health and socioeconomic burdens COVID-19 placed on families, communities of color also grappled with heightened xenophobia and racism. Yet, adolescents also found silver linings in the form of spending time with family and engaging in activities promoting relaxation and leisure. The present study examined parent-adolescent relationship quality (RQ) as a moderator of the relations of Latinx youth’s environment (i.e., racial-ethnic discrimination, COVID-19 stress, and COVID-19 silver linings) on their anxiety and depressive symptoms, both concurrently and six months later. Participants included 135 Latinx adolescents (*M*_age_ = 16.00, *SD* = 1.27; 59.3% female; 85.2% U.S.-born). Path analytic models revealed that youth discrimination experiences were positively associated with youth symptomatology, whereas COVID-19 silver linings and positive RQ were negatively associated with youth symptomatology. We also found that at mean and higher levels of negative RQ, discrimination experiences were positively associated with concurrent anxiety symptoms, suggesting that negative relationship features (e.g., conflict, pressure) exacerbated the effects of discrimination on youth anxiety. Therefore, stressors may predict youth symptomatology concurrently, but cultivating a positive parent-adolescent relationship and encouraging finding silver linings may bolster resilience in Latinx youth across time amidst adversity.

## 1. Introduction

In the United States, the COVID-19 pandemic was a chaotic period globally characterized by health-related concerns, job changes and financial stress, changes in family members’ routines (e.g., remote work, hybrid education) resulting from stay-at-home and shelter-in-place orders, loneliness and isolation, and great uncertainty in the family system. In the Latinx community, the pandemic placed unique pressures on youth and their families, including disparities in risk to health, hazardous working conditions, heightened economic adversity, experiences of racial-ethnic discrimination and xenophobia, increased parental stress, and greater mental health challenges (Acosta et al., 2021; Causadias & Neblett, 2024; Garcini et al., 2024; Villatoro et al., 2022). These sources of COVID-19 stress increased youth’s mental health symptoms (Villatoro et al., 2022).

Amid social and economic pressure as well as xenophobia and racism, some families found silver linings within the pandemic. For example, in April and May 2020, youth reported more quality time with families, reduced pressure from school and activities, and time for relaxation or creative pursuits (Silk et al., 2022). Within a primarily Latinx sample, higher youth-reported family functioning during April and May 2020 was related to lower youth-reported mental health symptoms (Penner et al., 2021). Experiencing silver linings early in the pandemic may have supported Latinx youth’s mental health, though the degree to which these protective effects persisted across the first year of the pandemic remains uncertain.

The bioecological model (Bronfenbrenner & Morris, 2006) indicates that youth functioning is nested within ecologies that have both proximal and distal impacts. “Accidents of history” in Bronfenbrenner’s chronosystem help us examine how stressful life events impact both the parent-adolescent relationship and youth development (Pérez-Edgar, 2021). Environmental stressors both within the macrosystem (i.e., COVID-19 policies, economy) and the microsystem (i.e., COVID-19 stress, experiences of discrimination) may disrupt critical face-to-face interactions between parents and adolescents and harm youth’s mental health. For example, Browne et al. (2021) found that disruption to the family’s economic and social needs in May 2020 was associated with less family cohesion and poorer mental health in children in July 2020. Heightened conflict within the parent-adolescent relationship may thus offset the effects of any silver linings from the pandemic, such as more time for leisure, and instead exacerbate the effects of COVID-related stressors and discrimination on youth symptomatology (Prime et al., 2020). However, higher warmth, affection, and support within the parent-adolescent relationship may serve as a buffer for COVID-19 stress and discrimination and support Latinx youth’s well-being and mental health. The present study examined whether parent-adolescent relationship quality moderated the associations of Latinx youth’s COVID-19 environment (i.e., discrimination, COVID-19 stress, COVID-19 silver linings) on their symptomatology, both concurrently and six months later.

### 1.1. COVID-19 Stress and Discrimination Within the Latinx Community

Racism and COVID-19 intersected in numerous ways, with the pandemic disproportionately affecting racially minoritized communities and amplifying experiences of discrimination. Latinx communities were among those most affected by the pandemic (Salgado de Snyder et al., 2021). Across all ages, Latinx individuals experienced disproportionately higher rates of COVID-19 infection, hospitalizations, ICU admissions, and in-hospital deaths when compared to non-Latinx White individuals (Acosta et al., 2021). Economic vulnerability, worries about one’s own health and the health of loved ones, changes in employment and family routines, increased childcare responsibilities, concerns about motivation and school performance amid remote learning, social isolation and changing relationships with family and peers, and increased exposure to discrimination and cyberbullying were among some of the stressors that youth faced during the pandemic (Lundström, 2022; Roche et al., 2022). Furthermore, Latinx youth reported being impacted by racism, the loss of income, an overload of information, and loneliness and boredom (Cortés-García et al., 2022). These stressors impacted critical developmental tasks for youth, such as identity formation and building autonomy, and disrupted sources of resilience, such as familial communication and coping (Santiago et al., 2020; Walsh, 2003).

Not surprisingly, the pandemic was a period of heightened anxiety and depressive symptoms among Latinx adolescents. In the United States in 2021, the previous year prevalence rate for depression disorders was 22.2% among Latinx youth aged 12 to 17 (SAMHSA, 2022). Within a sample of adolescents from public schools in Chicago, more Latinx youth scored in the clinical range for depression, generalized anxiety, and social anxiety than non-Latinx youth across the first two years of the pandemic, with girls and gender non-conforming youth reporting the highest levels of maladjustment (Polo et al., 2024). Latinx youth’s mental health was particularly impacted by stressors such as economic challenges and experiences of racism (Abreu et al., 2024). Exposure to illness-related events during the pandemic (e.g., illness due to COVID-19, loss of a loved one) was linked to distress among both Latinx and non-Latinx youth alike (D’Costa et al., 2021). Latinx youth also experienced greater internalizing and externalizing symptoms as a result of increased childcare responsibilities (Roche et al., 2022). Finally, in a previous analysis of this sample, COVID-19 stress during the height of the pandemic predicted greater depressive and anxiety symptoms six months later when youth reported low levels of problem-solving coping (Stein et al., 2024). Overall, multiple stressors were documented during the COVID-19 pandemic, leading to adverse consequences for Latinx youth’s mental health. However, further research is needed to better understand how and when COVID-19 stress and youth discrimination experiences contributed to their psychopathology symptoms and whether more adaptive parent-adolescent processes buffered these associations.

### 1.2. Sources of Resilience in Latinx Communities During the Pandemic

Despite the stressors that emerged due to the COVID-19 pandemic, cultural strengths within the Latinx community paved the pathway for silver linings. For example, between August and October 2020, Latinx youth reported getting along better with their siblings and spending more time with family members (Vélez-Grau et al., 2024). In fact, Latinx youth reported that school closures in spring 2020 were associated with more positive interactions with their siblings (Sun et al., 2021). A daily diary study found that when Latinx youth assisted their families on a given day or for longer than usual, these behaviors were linked to higher levels of same-day positive affect and lower levels of negative affect during the COVID-19 pandemic (Shen et al., 2024). For example, language brokering to help parents on a given day was associated with greater positive affect (Shen et al., 2024). Overall, Latinx youth and families reported increased time with family members and improved familial relationships, both initially during the pandemic (i.e., in April and May 2020; Cortés-García et al., 2022; Penner et al., 2021) and later in the first year (i.e., December 2020 to February 2021; Hammons et al., 2022). With regard to Latinx youth’s mental health, within a primarily Latinx sample, youth who reported more positive familial functioning (greater parental support and less parent-child conflict) also reported clinically significant reductions in symptoms during April and May 2020 (Penner et al., 2021). As the pandemic persisted across the first year, in this study’s sample, familial resilience (i.e., the ability of families to adapt in light of stress; Prime et al., 2020) was linked to fewer symptoms of depression (Stein et al., 2024). Qualitative research has also found that a strong connection with family members during the pandemic was beneficial for Latinx youth’s well-being (Cortés-García et al., 2022; Hammons et al., 2022). Additional research is needed to better understand the mechanisms by which COVID-19 positive experiences support Latinx youth’s mental health.

### 1.3. Parent-Adolescent Relationship Quality (RQ) as a Moderator

According to the bioecological model, face-to-face interactions between parents and adolescents are theorized to be the “engines of development” (i.e., proximal processes in the youth’s immediate environment) in which youth may learn about stressors and find resources for coping (Bronfenbrenner & Morris, 2006). However, if these interactions are disrupted during a stressful life event, adolescents’ fit to their immediate environment may be challenged, prompting either maladaptation or adjustment. Unfortunately, amid stay-at-home orders, there were plenty of opportunities for negative parent-adolescent interactions, especially if families were already vulnerable to experiencing conflict. Parenting stress intensified during the pandemic due to widespread family disruptions, such as increased childcare responsibilities, managing at-home schooling, job losses, interruptions to children’s routines, and hospitalizations (Adams et al., 2021). For Latinx families, economic and parenting stress were likely compounded by systemic inequities, such as limited access to healthcare and social support, language barriers, and ongoing experiences of discrimination, further straining family dynamics and contributing to parent-adolescent conflict. In a sample of Mexican-origin families, parents’ reports of economic hardship were associated with lower youth-reported parent-adolescent warmth and higher parent-adolescent conflict (Delgado et al., 2013). In low-income families, Latinx parents endorsing more social stressors also reported less child engagement (Cabrera et al., 2022). Similarly, a meta-analysis revealed that parenting stress during COVID-19 was linked to higher child externalizing and internalizing behaviors as well as lower parent-child RQ (Chung et al., 2024). These disruptions to the parent-adolescent relationship may exacerbate risk for youth symptomatology. For example, Magson et al. (2021) found that youth reporting more COVID-19 worries or greater conflict with their parents also exhibited increases in their anxiety and depressive symptoms from before the pandemic to two months into the pandemic. Therefore, negative parent-adolescent RQ may be a compounding risk factor that exacerbates the effects of youth discrimination experiences and COVID-19 stress on youth mental health.

On the other hand, amid heightened COVID-19 stress and discrimination experiences, positive parent-adolescent RQ may serve as a buffer, decreasing risk for internalizing symptoms. Early evidence from the pandemic revealed protective effects of positive parent-adolescent RQ on youth adjustment. For example, in a large sample of participants across five countries (i.e., Italy, the Philippines, Sweden, Thailand, and the United States), Skinner et al. (2021) found that higher youth-reported COVID-19 disruption to daily routines, work, and family life was associated with increases in youth internalizing symptoms in March 2020. However, these associations were attenuated if families reported markers of positive parent-adolescent RQ prior to the pandemic: higher levels of adolescent disclosure and supportive parenting and lower parent-adolescent conflict. Similarly, increases in positive parent-adolescent RQ during the pandemic were associated with decreases in youth mental health symptoms (i.e., depression, perceived stress, and emotion dysregulation) from fall 2019 to fall 2020 (Afriat et al., 2023). In recent work with adults, having more family health resources and higher familial cohesion, flexibility, and communication decreased the odds of mental health symptoms and supported well-being (Crandall et al., 2023; Lucia La Rosa et al., 2024). Further research is needed to examine whether positive parent-adolescent relationships buffer the effects of pandemic stressors (e.g., discrimination experiences, COVID-19 stress) on adolescents’ mental health, particularly across the first year of the pandemic. Latinx culture places a strong emphasis on warmth, closeness, and support in family relationships (e.g., familism), and this value is associated with positive youth adjustment through greater closeness to family members and greater perceived social support (Campos et al., 2014). Therefore, given the emphasis on family in Latinx culture, parent-adolescent RQ may be a critical source of resilience for Latinx families who support one another amidst adversity.

### 1.4. Present Study

This study aimed to examine whether parent-adolescent RQ during the pandemic moderated the associations of negative and positive environmental factors for Latinx youth (i.e., adolescents’ discrimination experiences, the severity of COVID-19 stress, and the number of COVID-19 silver linings) with their reports of their anxiety and depressive symptoms, both concurrently and six months later. In relation to main effects, we predicted that COVID-19 stress, adolescents’ discrimination experiences, and negative RQ would be positively associated with youth symptomatology, and that COVID-19 silver linings and positive RQ would be negatively related to youth symptomatology, both concurrently and across time. We also hypothesized that positive RQ would serve to buffer the effects of stressors (i.e., COVID-19 stress, adolescents’ discrimination experiences) and augment the effects of COVID-19 silver linings on youth symptomatology. On the other hand, we hypothesized that negative RQ would exacerbate risk from COVID-19 stress and discrimination and mitigate the relationship between COVID-19 silver linings and youth symptoms.

## 2. Materials and Methods

### 2.1. Participants

Participant recruitment occurred from October 2020 to September 2021 following approval from the Institutional Review Board. We used a non-probability sampling technique to recruit Latinx youth in the community. The study team distributed flyers outside of flea markets and food distributions and leveraged established community partnerships with Latinx-serving organizations to contact families. Youth eligible to be enrolled in the study had to be in middle or high school at the time of screening, self-identify as a Latinx adolescent between the ages of 13 and 18, and reside in North Carolina. If youth did not meet these criteria, they were excluded from the study.

Participants included 135 Latinx adolescents aged 13 to 18 (*M*_age_ = 16, *SD* = 1.27) who resided in North Carolina. This is an emerging immigrant destination in which youth and families may have fewer resources to deal with stressors compared to established immigrant communities (e.g., California, Texas). Of the adolescents in the sample, the majority identified as female (*n* = 80, 59.3%) and were born in the United States (*n* = 114, 84.4%). The remaining participants indicated they were foreign-born (*n* = 21) from Mexico (25%), Central America (30%; e.g., El Salvador, Guatemala), and South America (45%; e.g., Venezuela, Ecuador). Primary caregivers were mostly foreign-born from Mexico (*n* = 94, 69.6%) and Central and South America (*n* = 29, 21.5%), with only 8.9% (*n* = 12) born in the United States. Regarding household income, only 62% of adolescents knew their family household income (*n* = 84); over half of them (*n =* 57) reported an income less than $60,000. For more details about the sample, please see Stein et al. (2024).

### 2.2. Procedure

Participants were recruited for a longitudinal study to understand their experiences during the COVID-19 pandemic across six months. Youth were screened for their eligibility to participate on the phone by bilingual research assistants, and after obtaining parental consent, they received a Qualtrics link to complete a 40 min online survey. At the first timepoint (T1), adolescents reported on their discrimination experiences, COVID-19 stress and silver linings, parent-adolescent RQ, and mental health. The second timepoint (T2) was administered six months (*M* = 28.18 weeks, *SD* = 4 weeks) post-T1 with similar measures. We had a 77% participant retention rate (*n* = 104). Upon completion of each survey, participants received a $15 gift card and were entered into a drawing for a $100 gift card.

### 2.3. Measures

#### 2.3.1. Racial-Ethnic Discrimination

Using a shortened Experiences of Discrimination scale (Krieger et al., 2005), we assessed youth racial-ethnic discrimination experiences at T1. Youth used a Likert scale from 1 (*never*) to 4 (*four or more times*) to indicate how often they had experienced discrimination in school, at a restaurant or store, on the street or in a public setting, and by the police or in the courts. Item responses were averaged (α = 0.75), with higher scores indicating more frequent discrimination.

#### 2.3.2. COVID-19 Number of Silver Linings and Stress

We utilized an adapted version of the Responses to Stress Questionnaire-COVID-19, which included 11 items from the original measure (Compas, 2020; Connor-Smith et al., 2000) and 14 items developed for use in the Raising Grateful Children study (Hussong et al., 2022). The original measure is predictive of youth coping, and it has been validated with both adolescents and emerging adults (Connor-Smith et al., 2000). We used the Hussong et al. (2022) measure to assess the number of silver linings and the degree of stress that youth experienced during COVID-19. Participants indicated if they had experienced 11 silver linings (0 = no, 1 = yes) during the pandemic. We summed the total number (e.g., “I feel more creative since COVID-19”) that youth reported experiencing. To assess the stress experienced from COVID-19 negative events, participants were presented with 14 negative events (e.g., “Challenges at home or with others because of COVID-19”). Many of these events were similar to each other (e.g., asking about changes in routines, canceled plans, time away from friends), so we were less interested in a count of negative events and focused more on the stress experienced from these events. Youth rated how pleasant they found the experience to be using a scale from −4 (*extremely bad*) to 4 (*extremely good*). Given the lower base rates of pleasant responses for negative events, participants’ scores were recoded to range from 0 (*collapsing scores across extremely good to neither good or bad*) to 4 (*extremely bad*). Responses were then averaged (α = 0.92), with higher scores indicating experiencing more COVID-19 stress.

#### 2.3.3. Positive and Negative RQ

Adolescents reported on the quality of their parent-adolescent relationship at T1 via the Network of Relationships Inventory-Relationship Qualities Version (Buhrmester & Furman, 2008). The measure is composed of ten subscales of 3 items per scale for a total of 30 items. To assess negative RQ, we used the subscales assessing conflict (e.g., “How often do you and this person argue with each other?”), criticism (e.g., “How often does this person criticize you?”), dominance (e.g., “How often does this person get you to do things their way?”), exclusion (e.g., “How often does it seem like this person ignores you?”), and pressure (e.g., “How often does this person try to get you to do things that you don’t like?”). One exclusion item was omitted because it was perceived to be too confusing for participants. To assess positive RQ, we used the subscales assessing approval (e.g., “How often does this person seem really proud of you?”), companionship (e.g., “How often do you spend fun time with this person?”), emotional support (e.g., “How often do you depend on this person for help, advice, or sympathy?”), intimate disclosure (e.g., “How often do you share secrets and private feelings with this person?”), and satisfaction (e.g., “How happy are you with your relationship with this person?”). Adolescents rated these features of their parent-adolescent relationship using a scale from 1 (*never*) to 5 (*always*). Responses were averaged across the items for conflict, criticism, dominance, exclusion, and pressure to create an overall score, with higher values reflecting higher negative RQ (α = 0.89). Responses were averaged across the items for approval, companionship, emotional support, intimate disclosure, and satisfaction to create an overall score, with higher values reflecting higher positive RQ (α = 0.90).

#### 2.3.4. Adolescent Psychopathology Symptoms

Adolescent psychopathology symptoms were measured with the PROMIS Pediatric Short Form (Irwin et al., 2010) using separate scales for anxiety and depressive symptoms. The subscales were composed of 8 items each. Participants reported their anxiety (e.g., “I worried when I went to bed at night”) and depressive symptoms (e.g., “It was hard for me to have fun”) at T1 and T2 using a Likert scale from 1 (*never*) to 5 (*almost always*). Scores were averaged separately, with higher scores indicating either higher anxiety or depressive symptoms. The scales displayed good internal consistency at both T1 (anxiety: α = 0.93, depressive symptoms: α = 0.95) and T2 (anxiety: α = 0.92, depressive symptoms: α = 0.96).

### 2.4. Data Analytic Plan

We ran eight path analytic models testing parent-adolescent RQ (i.e., positive or negative) as a moderator of the effects of adolescents’ COVID-19 environmental context (i.e., discrimination experiences, COVID-19 stress, and COVID-19 silver linings) in predicting youth symptomatology. We ran separate models assessing relations with youth anxiety and depressive symptoms, both concurrently and six months later. Power analyses conducted for linear multiple regression in G*Power (Faul et al., 2007) revealed that a sample of 135 youth could be used to detect a medium to large effect size (f^2^ ≥ 0.17). Only 92 youth reported on their symptomatology at T2, so we used a full information maximum likelihood approach to handle missing data and estimate each parameter using all available information. Youth with missing data were younger (*M*_age_ = 15.60 years) than youth with complete data (*M*_age_ = 16.18 years), *t*(67.46) = 2.36, *p* = 0.02. There were no other significant differences in attrition rates based on youth gender, youth nativity, parent education, and family income. Since youth age was unrelated to youth symptomatology, to preserve power, we did not include this as a covariate in the models. Looking at our study variables at T1, there were no significant differences in attrition rates based on adolescent-reported discrimination, COVID-19 stress, COVID-19 silver linings, positive RQ, negative RQ, and anxiety and depressive symptoms at T1. 

Models were run using the *lavaan* package (Rosseel, 2012) in RStudio version 2024.12.1 (RStudio Team, 2025). All variables were standardized and mean-centered before computing interaction variables. Model fit was assessed using a non-significant chi-square, comparative fit and Tucker-Lewis indices (i.e., CFI and TLI, respectively) indicating values of 0.95 or above, a root-mean-square error of approximation (RMSEA) value below 0.06, and a standardized root-mean-square residual (SRMR) value below 0.08 (Hu & Bentler, 1999). Any significant interaction terms were probed post-hoc using Johnson-Neyman plots to assess for what values of the moderator the association between the predictor and outcome was significant at *p* < 0.05.

## 3. Results

### 3.1. Preliminary Analyses

We report the descriptive statistics and correlations for the study variables in Table 1. On average, adolescents in this sample reported moderate levels of discrimination (*M* = 1.76/4), and these were weakly positively correlated with COVID-19 stress and negative parent-adolescent RQ and moderately positively correlated with anxiety and depressive symptoms at T1. On average, adolescents also reported moderate levels of COVID-19 stress (*M* = 2.01/4), which were positively correlated with anxiety and depressive symptoms at T1. Adolescents endorsed experiencing an average of 4.56 out of 11 COVID-19 silver linings; this was negatively correlated with youth T1 anxiety and depressive symptoms and youth T2 anxiety symptoms. Youth anxiety and depressive symptoms were highly correlated at both T1 and T2.

### 3.2. Positive RQ Models

#### 3.2.1. Concurrent Models

**Figure 1:**
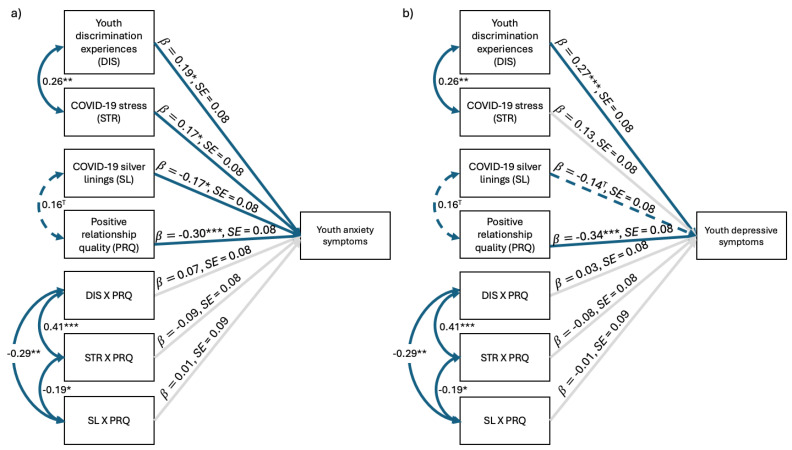
Positive relationship quality moderating the effects of environmental factors on concurrent (**a**) youth anxiety and (**b**) depressive symptoms. *N* = 135. Standardized regression coefficients depicted. Solid blue lines represent significant associations, dashed blue lines indicate marginally significant associations, and light gray lines represent nonsignificant associations. ^T^ *p* < 0.10, * *p* < 0.05, ** *p* < 0.01, *** *p* < 0.001.

The anxiety model fit the data well (Figure 1a), χ^2^(16) = 10.66, *p* = 0.83, CFI = 1.00, TLI = 1.14, RMSEA = 0.00, SRMR = 0.04, and explained significant variance in youth T1 anxiety (22%). As hypothesized, discrimination, *b* = 0.19, *SE* = 0.08, *z* = 2.36, *p* = 0.02, and COVID-19 stress, *b* = 0.17, *SE* = 0.08, *z* = 2.04, *p* = 0.04, were both positively associated with youth anxiety. Additionally, COVID-19 silver linings, *b* = −0.17, *SE* = 0.08, *z* = −2.12, *p* = 0.03, and positive RQ, *b* = −0.30, *SE* = 0.08, *z* = −3.86, *p* < 0.001, were negatively associated with youth anxiety symptoms. There were no statistically significant interaction effects.

The depressive symptoms model fit the data well (Figure 1b), χ^2^(16) = 10.65, *p* = 0.83, CFI = 1.00, TLI = 1.13, RMSEA = 0.00, SRMR = 0.04, and explained significant variance in youth T1 depressive symptoms (27%). As hypothesized, discrimination was positively associated with youth depressive symptoms, *b* = 0.27, *SE* = 0.08, *z* = 3.50, *p* < 0.001, whereas positive RQ was negatively related to youth symptoms, *b* = −0.34, *SE* = 0.08, *z* = −4.49, *p* < 0.001. Contrary to what we expected, COVID-19 silver linings were only marginally associated with youth depressive symptoms, *b* = −0.14, *SE* = 0.08, *z* = −1.83, *p* = 0.07, and COVID- 19 stress was unrelated to youth depressive symptoms. There were no statistically significant interaction effects.

#### 3.2.2. Longitudinal Models

**Figure 2:**
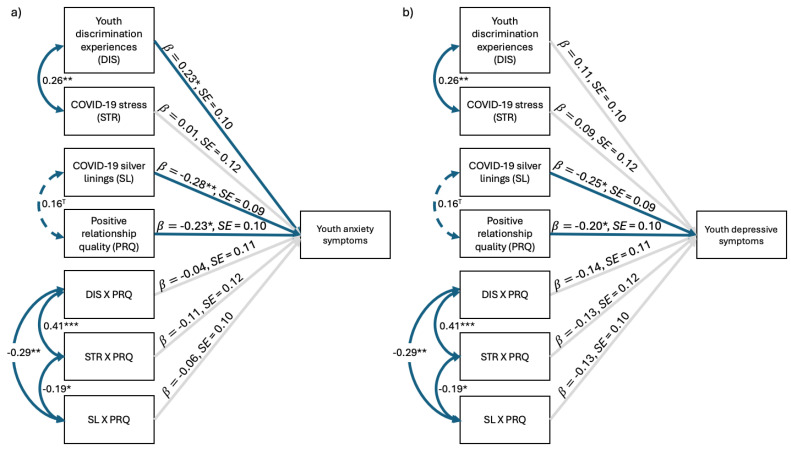
Positive relationship quality moderating the effects of environmental factors on (**a**) youth anxiety and (**b**) youth depressive symptoms six months later. *N* = 135. Standardized regression coefficients depicted. Solid blue lines represent significant associations, dashed blue lines indicate marginally significant associations, and light gray lines represent nonsignificant associations. ^T^ *p* < 0.10, * *p* < 0.05, ** *p* < 0.01, *** *p* < 0.001.

The anxiety model fit the data well (Figure 2a), χ^2^(16) = 10.65, *p* = 0.83, CFI = 1.00, TLI = 1.17, RMSEA = 0.00, SRMR = 0.04, and explained significant variance in youth T2 anxiety symptoms (22%). As hypothesized, like in the concurrent model, discrimination was positively associated with youth anxiety, *b* = 0.23, *SE* = 0.10, *z* = 2.34, *p* = 0.02. Additionally, COVID-19 silver linings, *b* = −0.28, *SE* = 0.09, *z* = −2.99, *p* = 0.003, and positive RQ, *b* = −0.23, *SE* = 0.10, *z* = −2.35, *p* = 0.02, once again exhibited significant negative relations with youth symptoms. COVID-19 stress was no longer related to youth anxiety six months later. There were no statistically significant interaction effects.

The depressive symptoms model fit the data well (Figure 2b), χ^2^(16) = 10.65, *p* = 0.83, CFI = 1.00, TLI = 1.18, RMSEA = 0.00, SRMR = 0.04, and explained significant variance in youth T2 depressive symptoms (19%). Surprisingly, discrimination and COVID-19 stress were unrelated to youth symptomatology. However, like the concurrent model, positive RQ exhibited a significant negative relation with youth symptoms, *b* = −0.20, *SE* = 0.10, *z* = −2.02, *p* = 0.04. COVID-19 silver linings also emerged as a predictor of youth depressive symptoms, *b* = −0.25, *SE* = 0.09, *z* = −2.64, *p* = 0.01. There were no significant interaction effects.

### 3.3. Negative RQ Models

#### 3.3.1. Concurrent Models

**Figure 3:**
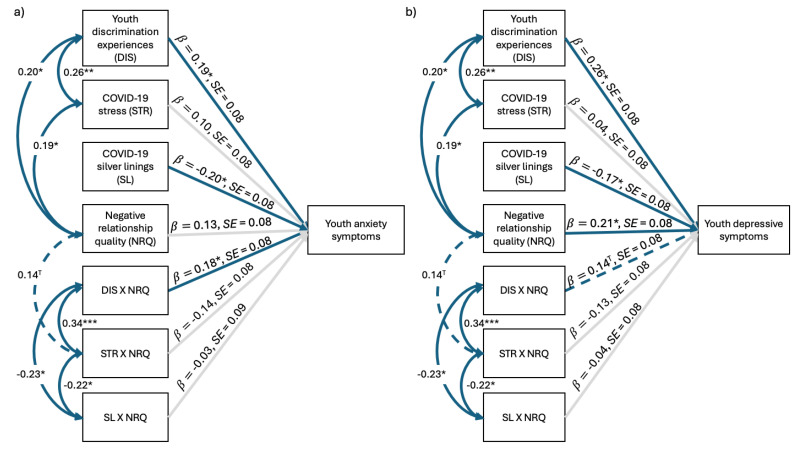
Negative relationship quality moderating the effects of environmental factors on concurrent (**a**) youth anxiety and (**b**) youth depressive symptoms. *N* = 135. Standardized regression coefficients depicted. Solid blue lines represent significant associations, dashed blue lines indicate marginally significant associations, and light gray lines represent nonsignificant associations. ^T^ *p* < 0.10, * *p* < 0.05, ** *p* < 0.01, *** *p* < 0.001.

The anxiety model fit the data well (Figure 3a), χ^2^(14) = 9.47, *p* = 0.80, CFI = 1.00, TLI = 1.16, RMSEA = 0.00, SRMR = 0.05, and explained significant variance in youth T1 anxiety symptoms (16%). As hypothesized, once again, discrimination was positively related to youth anxiety symptoms, *b* = 0.19, *SE* = 0.08, *z* = 2.22, *p* = 0.03, whereas COVID-19 silver linings were negatively associated with youth symptoms, *b* = −0.20, *SE* = 0.08, *z* = −2.41, *p* = 0.02. The interaction between youth discrimination experiences and negative RQ was also a statistically significant predictor of youth anxiety, *b* = 0.18, *SE* = 0.08, *z* = 2.10, *p* = 0.04. No other main effects or interaction effects were statistically significant.

**Figure 4:**
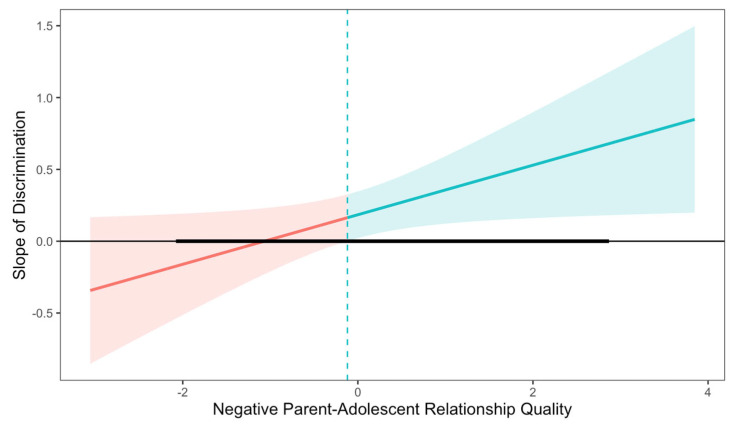
Johnson–Neyman plot describing the regions of significance for the interaction between racial-ethnic discrimination and negative parent-adolescent relationship quality in predicting youth concurrent anxiety symptoms. The bolded line represents the range of observed values for negative parent-adolescent relationship quality. The blue region represents the range of values for negative parent-adolescent relationship quality for which the slope of discrimination is significantly associated (*p* < 0.05) with youth symptoms; the red represents the region of non-significance. When negative relationship quality is greater than −0.12, racial-ethnic discrimination is positively associated with concurrent youth anxiety symptoms.

We used a post-hoc Johnson–Neyman plot to describe for which values of negative RQ the association was significant between discrimination and youth T1 anxiety symptoms (see Figure 4). At mean levels of negative RQ or higher (above −0.12), discrimination was positively related to youth T1 anxiety. Aligning with our hypothesis, mean levels of negative RQ or higher exacerbated the detrimental effect of discrimination on youth anxiety symptoms. However, there was no effect of discrimination on youth anxiety at lower levels of negative RQ.

The depressive symptoms model fit the data well (Figure 3b), χ^2^(14) = 9.57, *p* = 0.79, CFI = 1.00, TLI = 1.14, RMSEA = 0.00, SRMR = 0.05, and explained significant variance in youth T1 depressive symptoms (19%). As hypothesized, discrimination was positively related to youth depressive symptoms, *b* = 0.26, *SE* = 0.08, *z* = 3.12, *p* = 0.002, as was negative RQ, *b* = 0.21, *SE* = 0.08, *z* = 2.56, *p* = 0.01. Meanwhile, COVID-19 silver linings was negatively associated with youth symptoms, *b* = −0.17, *SE* = 0.08, *z* = −2.18, *p* = 0.03. COVID-19 stress was unrelated to youth outcomes. There were no significant interaction effects.

#### 3.3.2. Longitudinal Models

**Figure 5:**
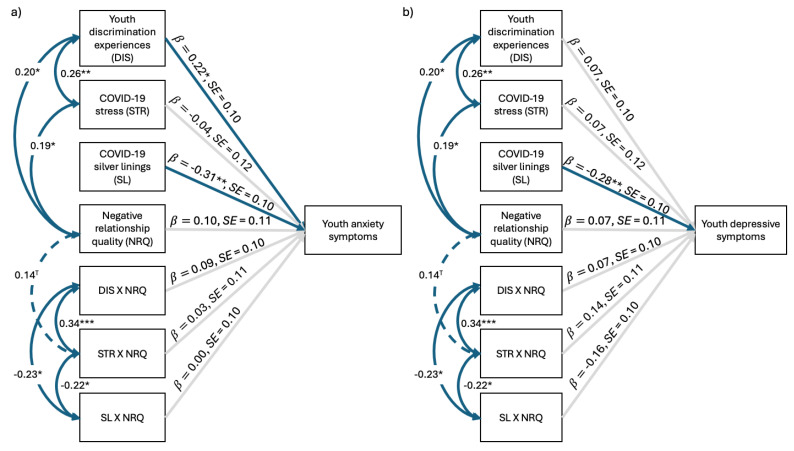
Negative relationship quality moderating the effects of environmental factors on (**a**) youth anxiety and (**b**) depressive symptoms six months later. *N* = 135. Standardized regression coefficients depicted. Solid blue lines represent significant associations, dashed blue lines indicate marginally significant associations, and light gray lines represent nonsignificant associations. ^T^ *p* < 0.10, * *p* < 0.05, ** *p* < 0.01, *** *p* < 0.001.

The anxiety model fit the data well (Figure 5a), χ^2^(14) = 9.46, *p* = 0.80, CFI = 1.00, TLI = 1.18, RMSEA = 0.00, SRMR = 0.05, and explained significant variance in youth T2 anxiety (17%). As hypothesized, like in the concurrent model, discrimination was positively associated with youth anxiety, *b* = 0.22, *SE* = 0.10, *z* = 2.14, *p* = 0.03. COVID-19 silver linings once again exhibited a significant negative relation with youth symptoms, *b* = −0.31, *SE* = 0.10, *z* = −3.20, *p* = 0.001. COVID-19 stress was not related to youth anxiety six months later. There were no other statistically significant main effects or interaction effects.

The depressive symptoms model fit the data well (Figure 5b), χ^2^(14) = 9.46, *p* = 0.80, CFI = 1.00, TLI = 1.18, RMSEA = 0.00, SRMR = 0.04, and explained significant variance in youth T2 depressive symptoms (17%). Once again, surprisingly, discrimination, COVID-19 stress, and negative RQ were unrelated to youth symptomatology. However, COVID-19 silver linings was negatively associated with youth T2 depressive symptoms, *b* = −0.28, *SE* = 0.10, *z* = −2.92, *p* = 0.003. There were no significant interaction effects.

## 4. Discussion

During a global stressful life event like COVID-19, the bioecological model (Bronfenbrenner & Morris, 2006) posits that environmental factors play an important role in shaping adolescent mental health and that more proximal relational processes (i.e., parent-adolescent relationship quality) may moderate their effects on youth mental health. The present study examined whether parent-adolescent RQ moderated the associations of COVID-19 stress, COVID-19 silver linings, and ethnic-racial discrimination with adolescent symptomatology, both concurrently and six months later. Notably, positive RQ was negatively associated with youth anxiety and depressive symptomatology at both timepoints, whereas negative RQ was only positively associated with youth concurrent depressive symptoms. We also found that at mean and higher levels of negative RQ, discrimination experiences were positively associated with concurrent anxiety symptoms, suggesting that negative relationship features (e.g., conflict, pressure) exacerbated the effects of discrimination on youth anxiety. Therefore, stressors may support youth symptomatology concurrently, but cultivating a positive parent-adolescent relationship and encouraging finding silver linings during times of stress may bolster long-term resilience in Latinx youth.

### 4.1. Positive RQ Supportive of Lower Symptomatology

A preponderance of literature demonstrates the beneficial effects of positive RQ on adolescents’ psychological adjustment (e.g., Khaleque, 2013). In this study, a composite for positive relationship quality reflecting the degree of approval, companionship, emotional support, intimate disclosure, and satisfaction within the parent-adolescent relationship was promotive of lower symptomatology, even amidst adversity. Latinx culture places an emphasis on strong support and cohesion among family members (Campos et al., 2014). Prior work in this sample found that parental support was positively associated with perceptions of familial resilience during the pandemic (Lobo et al., 2025). Positive family functioning within a primary Latinx sample has been associated with a reduction in symptoms in mid-April 2020 (Penner et al., 2021). Our findings extend this research to show that a positive parent-adolescent relationship may have supported better adolescent mental health across the first year of the pandemic, regardless of youth COVID-19 stress or discrimination experiences.

Higher positive RQ may have supported familial coping processes (Santiago et al., 2020). Some qualitative work shows that many Latinx adolescents did not discuss their COVID-19 worries with their parents, possibly in an effort to not add further discomfort to the family during this stressful life event; however, time spent connecting with family members, sharing meals, and participating in cultural activities like attending virtual church services were resources used to strengthen family relationships and support youth coping (Vélez-Grau et al., 2024). During the pandemic, finding positive activities to do together helped build a sense of trust, togetherness, cohesion, and happiness, supporting familial resilience (Gayatri & Irawaty, 2022; Walsh, 2003).

Positive RQ may also foment interactions that bolster youth positive affect and emotion regulation. In a daily diary study, Mercado et al. (2019) found that Mexican-origin parent-adolescent dyads who reported getting along more were more likely to engage in emotion coregulation of their daily happiness (i.e., the process of shaping one another’s emotional states towards happiness). In relation to emotion regulation, in an ethnically racially diverse sample, supportive parent-adolescent relationships were associated with higher adolescent prosocial behavior and lower aggression and depressive symptoms through positive effects on adolescents’ anger regulation (Ratliff et al., 2023). In turn, more prosocial behavior and lower aggression may further support positive RQ. Further research is needed to understand how positive relationship features may foment parent-adolescent coregulation processes around positive emotions that may support Latinx youth’s coping and regulation, even amidst adversity. Our findings indicate that amidst a stressful life event like the pandemic, positive RQ is an important target for clinicians that may be a source of resilience for Latinx youth.

### 4.2. Negative RQ and COVID-19 Silver Linings

Negative parent-adolescent RQ may disrupt adaptive socialization processes within the family and is associated with poor youth adjustment (Son et al., 2024). Given the importance of face-to-face interactions for youth development (Bronfenbrenner & Morris, 2006), we anticipated that negative RQ would be harmful for youth symptomatology by itself and exacerbate the detrimental effects of more distal stressors (e.g., discrimination experiences, COVID-19 stress) as a moderator. In terms of main effects, when accounting for the other stressors, negative parent-adolescent RQ (i.e., conflict, criticism, dominance, exclusion, and/or pressure) was associated with concurrent depressive symptoms. Youth who were forced to stay at home and were experiencing higher parenting stress and strained familial relationships may have felt hopeless and lonely.

Negative RQ did not exhibit long-term effects on youth symptomatology. There were only weak correlations with T2 anxiety and depressive symptoms, which were not statistically significant in regression models. As the pandemic persisted, the external stressors resulting from macrosystem changes that were evoking higher negative RQ may have been alleviated (e.g., parent finds new position after job loss; youth connects with peers or engages in extracurricular activities through online platforms). Other factors may have mitigated the effects of negative RQ. For example, after strained familial dynamics around sharing a space due to stay-at-home orders, families may have settled into routines; maintaining routines buffered the impact of the pandemic on youth mental health (Ren et al., 2021) and familial resilience (Bates et al., 2021). Donker et al. (2025) found that families who were able to decrease their negative interactions also observed decreases in youth mental health symptoms from fall 2020 to spring 2021. Youth coping has also been shown to buffer the effects of family conflict on youth adjustment (Wadsworth & Compas, 2002); youth may have developed coping mechanisms (e.g., gained access to online mental health resources or therapy, connected to peer support through social media) or found COVID-19 silver linings (e.g., time for journaling and pursuing hobbies) that supported their mental health.

Notably, across all models, endorsing more COVID-19 silver linings was associated with lower symptomatology concurrently and six months later, and, unexpectedly, parent-adolescent RQ did not moderate this effect. Youth in this study reported spending more time with family (68%), pursuing hobbies (64%), finding ways to help people (50%), and getting along with siblings (50%). Similarly, in one qualitative study, Latinx youth reported finding silver linings by using the time to renew interest in activities (e.g., journaling, painting) and discover new hobbies, as well as reflect on important questions about their goals, family, and spirituality during quarantine (Vélez-Grau et al., 2024), bolstering identity development. In a daily diary study, assisting their families was linked to higher levels of same-day positive affect for Latinx youth (Shen et al., 2024). Amidst an uncontrollable stressor like the pandemic, these silver linings may have reflected an active coping strategy, allowing youth to *shift* their perspective and reframe the changes resulting from COVID-19 as opportunities for growth and *persist* in pursuing their goals and helping their families amidst adversity; shift-and-persist coping is a culturally-consonant form of coping in Latinx families associated with higher familial resilience during the pandemic (Lobo et al., 2025) and better mental health in Latinx youth amid discrimination (Christophe et al., 2019). It is posited that this form of coping is particularly effective when individuals are faced with uncontrollable stressors (e.g., economic strain, discrimination) (Chen & Miller, 2012) and was reported by adolescents during the pandemic (Lehmann et al., 2024). In a community sample of adults, higher cognitive reframing during the pandemic was related to a higher engagement in activities for pleasure, which in turn was related to higher reported cognitive and affective happiness (Gutiérrez-Cobo et al., 2021).

Surprisingly, higher negative RQ did not mitigate these effects: COVID-19 silver linings were supportive of youth mental health regardless of negative RQ. At lower levels of negative RQ, other protective factors may already have been supporting adolescent mental health (e.g., parental warmth, family cohesion; e.g., Penner et al., 2021). On the other hand, at higher negative RQ, these silver linings may have afforded youth an ability to focus on their own needs and engage in hobbies or creative pursuits, offering avoidance or respite from engaging in a negative parent-adolescent relationship. Youth may have also cognitively reframed their strained parent-adolescent dynamics by attributing them to uncontrollable, external stressors or seeing them as situational, and pursued their interests as a way to bolster their mental health (Lehmann et al., 2024). These silver linings may have also supported youth positive affect (Gutiérrez-Cobo et al., 2021), which may have helped reduce youth involvement in negative parent-adolescent interactions.

### 4.3. The Detrimental Effects of Racial-Ethnic Discrimination

Overwhelmingly, the literature points to the detrimental impact of racial-ethnic discrimination on youth symptomatology (e.g., Benner et al., 2018). Following this literature, racial-ethnic discrimination was associated with youth T1 anxiety and depressive symptoms and youth T2 anxiety symptoms. Building on prior work, our research also highlights that higher negative parent-adolescent RQ (i.e., conflict, criticism, dominance, exclusion, and/or pressure) may exacerbate the effects of an external stressor like racial-ethnic discrimination on concurrent youth anxiety symptoms. Vélez-Grau et al. (2024) found that youth experiencing strained familial relationships during the pandemic reported an increased sense of isolation and disconnection. Thus, negative parent-adolescent RQ may also disrupt familial coping processes, shown to support youth coping and mental health in Latinx families (Santiago et al., 2020; Santiago et al., 2021). Prior work in Latinx communities has highlighted that families often support youth in coping with racial-ethnic discrimination (Martin Romero et al., 2022). However, when the parent-adolescent relationship is already negative, Latinx youth may be reluctant to share about their discrimination experiences and may instead choose self-silence in order to maintain harmony in interpersonal interactions (i.e., simpatía; Acevedo et al., 2020); this may diminish their capacity to cope with this stressor, contributing to higher anxiety symptoms.

As the pandemic persisted across the first year, racial-ethnic discrimination was associated with youth anxiety six months later, not depressive symptoms; unexpectedly, negative RQ no longer moderated this association. Youth experiencing discrimination may have become more anxious about the possibility of experiencing further exclusionary or threatening behavior or felt unsure about how to respond or cope without continued access to peer support. If youth’s discrimination experiences were pervasive or severe, or if they were being harmed through the experiences of others in the family (e.g., vicarious racism; Martin Romero & Stein, 2023), then discrimination may have continued to impact youth symptomatology regardless of the quality of the parent-adolescent relationship. For example, at a systemic level, qualitative research showed that Latinx adolescents were aware that their community was being disproportionately impacted by the pandemic due to racism, leading to major health and financial disparities (Cortés-García et al., 2022). Interpersonally, despite in-person school activities halting, youth may have also continued to experience discrimination online due to stigmatized attributes (e.g., skin tone, accent, language ability), perceived affiliations (e.g., immigrant), and stereotypes (e.g., assumed unintelligent or criminal) (Portillo et al., 2022; Torres et al., 2022). Amidst higher discrimination, higher negative RQ may have prompted some youth to seek out other sources of social support (e.g., friends, siblings) or develop their own coping skills, mitigating the effects of this stressor as a moderator.

One possible explanation for a lack of findings with depressive symptoms could be changes in youth’s exposure to perpetrators of discrimination. Some youth may have had less in-person exposure to perpetrators of discrimination, like peers, due to canceled school events and activities, thus contributing to lower symptoms. However, other adolescents may have had increased exposure. Exposure to discrimination via social media increased adolescents’ depressive symptoms (Tao & Fisher, 2022). Given increased time spent with families, youth may also have received more parental socialization messages boosting their ethnic pride as well as preparing them for bias and supporting their coping (i.e., racial-ethnic socialization; Ayón et al., 2020), mitigating the effects of this stressor on feelings of hopelessness or loneliness. Longitudinal research is needed to understand the complex relations between parental racial-ethnic socialization and adolescent coping with discrimination across time, and the moderating effects of parent-adolescent RQ.

### 4.4. The Impacts of COVID-19 Stress on Youth Symptomatology

This study found that COVID-19 stress was positively associated with concurrent anxiety symptoms in Latinx youth, and parent-adolescent RQ did not moderate this association. Prior research conducted during the first year of the pandemic found that in a sample of Latinx youth ages 11 through 18, higher COVID-19 health, economic, and social impacts were associated with higher anxiety symptoms, depressive symptoms, and sleep disturbances (Kuhlman et al., 2023). In this sample, the majority of youth reported experiencing changes in their routines (86.5%), an inability to spend time with friends (82.0%), and uncertainty about the future (85.7%) as COVID-19 stressors. Within a sample of primarily White, non-Hispanic youth (83%), Hawes et al. (2021) found that similar concerns about being confined at home (e.g., having a limited social life, experiencing cabin fever) in the spring of 2020 were positively associated with generalized anxiety in a large sample of adolescents, but not depressive symptoms. Study findings suggest these effects may have persisted across the first year: Latinx youth may have been anxious about the uncertainty around the pandemic and experienced a loss of control, distinct markers of anxiety symptoms. Given that these associations were concurrent, adolescents’ anxiety may have also inhibited their coping and increased their perception of the unpleasantness of COVID-19 stressors. However, six months later, youth may have fallen into routines with their families that mitigated against symptoms resulting from uncertainty (Ren et al., 2021). Circumstances may have also changed, alleviating COVID-19 stress for families (e.g., youth finding a job to help support their family).

Contrary to what we expected, negative RQ did not exacerbate these effects. Drawing from the bioecological model (Bronfenbrenner & Morris, 2006), one possible explanation is that youth also experiencing harmful parent-adolescent interactions may have developed both maladaptive coping mechanisms (e.g., Francisco et al., 2016) and heightened symptoms as a result. As such, there may be a ceiling effect on youth distress: additional stressors from the pandemic may not be increasing their symptoms further, or youth may have blunted emotional responses to them (Spies et al., 2011). Qualitative research revealed that Latinx youth experiencing strained familial relationships during the pandemic would also limit family interactions (e.g., stay in their room), which may have also protected them from being exposed to other COVID-19 stressors (Vélez-Grau et al., 2024).

Surprisingly, COVID-19 stress was also unrelated to youth T1 and T2 depressive symptoms. Prior work in this sample showed relations between COVID-19 stress and youth depressive symptoms (Stein et al., 2024); however, this work did not consider the roles of other variables that may have been more salient for youth symptoms during this period, such as their own experiences of discrimination and parent-adolescent relationship quality. Prior longitudinal work in a community sample also found that trajectories of increasing depressive symptoms across the first 8 months of the pandemic may be affected by a complex moderation by youth gender: boys demonstrated increases in their depressive symptoms, whereas girl’s depressive symptoms only increased if they spent more time online on social media or playing social video games (Liu et al., 2022). Rumination about COVID-19 stressors with peers may have moderated this association, though future research is needed to test this with larger samples.

### 4.5. Strengths, Limitations, and Future Directions

Much of the literature has examined the effects of the onset of the pandemic on adolescents’ psychopathology symptoms, particularly in White populations. However, Latinx youth were disproportionately impacted during the pandemic. A major strength of this study was the consideration of multiple aspects of Latinx youth’s environment, both positive (i.e., COVID-19 silver linings) and negative (i.e., discrimination experiences, COVID-19 stress) that may have impacted their anxiety and depressive symptoms six months later as the pandemic persisted across the first year. Given the importance of the family within Latinx communities (Campos et al., 2014), the study findings present an important step forward in understanding how the quality of the parent-adolescent relationship, measured across a number of features, may serve as a source of resilience for Latinx youth’s mental health amid a stressful life event.

However, the study included some noteworthy limitations. In accordance with other work conducted during the pandemic (Yu et al., 2022), this study included a smaller sample size and had an attrition rate of 77% across six months; as such, we did not have the statistical power to detect small effect sizes in our models, which may have precluded our ability to detect moderation effects. We found no mean differences in study variables at T1 between those who participated at T2 and those who did not. To account for the missing data, we used an analytic approach (i.e., full information maximum likelihood) that uses all available information from participants to estimate parameters. However, youth experiencing more stressors (i.e., discrimination experiences, COVID-19 stress), more strained dynamics, and/or higher symptoms at T2 may have chosen not to participate; this may have affected our ability to detect main effects across time for these stressors and negative RQ and affected the generalizability of the findings.

The majority of participants were born in the United States (84%), and the geographic concentration of the sample in North Carolina may have limited the generalizability of these results. Data were collected in an emerging immigrant destination offering fewer resources to families than a more established immigrant community. Therefore, families may have had fewer resources or support for navigating the challenges of the pandemic, exacerbating COVID-19 stress. Our findings are limited to youth living in similar contexts and may not extend to other populations. Findings must be replicated in studies with larger, diverse samples of Latinx youth from a variety of contexts (i.e., Latinx immigrant youth, rural versus urban areas, established versus emerging immigrant communities).

Families also completed the first survey during a window of eleven months (i.e., October 2020 to September 2021), during which we observed important changes in education (e.g., remote versus hybrid classrooms), policy (e.g., mask mandates, social distancing), and health (e.g., access to vaccines); however, survey completion date was not associated with any study variables. This period entailed significant societal disruption, and findings may generalize to similar stressful life events (e.g., natural disasters, epidemics).

Lastly, though we collected these data while the pandemic was ongoing, rather than using retroactive reporting, all variables were assessed using youth reports and associations may reflect self-report biases or shared method variance. For example, youth may be more reluctant to report negative family dynamics or higher mental health symptoms. Utilizing reports from multiple family members may have increased our understanding of family experiences of COVID-19 stressors and silver linings. Another limitation is that given that the pandemic had a sudden onset and was time-limited, we could not further validate the measure of COVID-19 stress and silver linings in another sample; however, as expected, correlations showed that COVID-19 stress was positively associated with concurrent youth symptoms, and COVID-19 silver linings was negatively associated with symptoms concurrently and six months later.

Despite study limitations, this study captured many markers of the parent-adolescent relationship during a stressful life event characterized by sociopolitical and economic instability and significant disruptions to the family system. Supporting prior research, COVID-19 stress was associated with higher concurrent youth anxiety. Policymakers and educators should attempt to minimize disruptions to youth by advocating for policies that encourage connection (e.g., virtual meetings for social clubs) or strong mitigation measures (e.g., masking) that reduce disruptions for communities of color. In line with theory (Bronfenbrenner & Morris, 2006), we found that negative RQ exacerbated the effects of adolescents’ discrimination experiences on their anxiety symptoms. However, positive RQ and COVID-19 silver linings emerged as important sources of resilience long-term, regardless of youth-reported discrimination experiences and COVID-19 stress. Therefore, these may be important targets for interventions that support positive face-to-face interactions between parents and adolescents as well as youth coping amidst a stressful life event. These findings point to the importance of clinicians supporting Latinx youth mental health through bolstering familial connection and coping, reducing parent-adolescent conflict, and helping youth reframe stressful life events as opportunities for growth. Finally, further research is needed to examine how dynamic changes in adolescents’ environment and more proximal parent-adolescent processes across the onset and duration of stressful life events are related to Latinx youth’s adjustment. Given the findings suggesting the importance of positive RQ, research is needed to understand how this may intersect with parent messages building youth’s ethnic pride and racial-ethnic identity (Ayón et al., 2020) and coping skills (Wadsworth & Compas, 2002) to support their mental health amidst adversity.

## Figures and Tables

**Table 1:** Descriptive statistics for study variables.

Variable	1	2	3	4	5	6	7	8	9	10	11	*n*	*M*	*SD*
1. Gender (T1)	1											132	0.39	0.49
2. Age (T1)	−0.28 **	1										134	16.00	1.27
3. Nativity (T1)	0.10	−0.11	1									135	0.84	0.36
4. Racial-ethnic discrimination (T1)	−0.21 *	0.14	−0.02	1								133	1.76	0.71
5. COVID-19 stress (T1)	−0.13	0.12	−0.09	0.26 **	1							130	2.01	1.01
6. COVID-19 silver linings (T1)	0.13	−0.03	0.01	−0.14	−0.10	1						133	4.52	2.58
7. Positive relationship quality (T1)	−0.02	−0.01	−0.07	−0.02	0.11	0.16	1					133	3.24	0.83
8. Negative relationship quality (T1)	−0.02	0.00	0.11	0.21 *	0.17 ^T^	−0.13	−0.30 **	1				133	2.44	0.70
9. Anxiety symptoms (T1)	−0.22 *	0.16 ^T^	−0.07	0.26 **	0.21*	−0.25 **	−0.31 ***	0.21 *	1			134	51.99	12.64
10. Depressive symptoms (T1)	−0.19 *	0.13	0.00	0.33 ***	0.18*	−0.24 *	−0.35 ***	0.28 **	0.83 ***	1		134	53.45	11.91
11. Anxiety symptoms (T2)	−0.28 *	0.12	−0.03	0.25 *	0.14	−0.34 **	−0.27 *	0.21 *	0.62 ***	0.54 ***	1	92	52.01	12.31
12. Depressive symptoms (T2)	−0.14	0.14	−0.07	0.15	0.14	−0.31 **	−0.24 *	0.21 *	0.46 ***	0.47 ***	0.80 ***	92	53.52	11.43

Note. N = 135. T1 = time 1, T2 = time 2. Gender was coded as a dichotomous variable (0 = female, 1 = male), as was nativity status (0 = foreign-born, 1 = U.S.-born). Statistically significant correlations are bolded. ^T^ *p* < 0.10, * *p* < 0.05, ** *p* < 0.01, *** *p* < 0.001.

## Data Availability

The data presented in the study and analysis code are available in OSF at https://doi.org/10.17605/OSF.IO/T5V2Q.
